# Transgenerational transfer of gene-modified T cells

**DOI:** 10.1186/s40425-019-0657-2

**Published:** 2019-07-15

**Authors:** Cormac Cosgrove, Emilia R. Dellacecca, Joost H. van den Berg, John B. Haanen, Michael I. Nishimura, I. Caroline Le Poole, Hans E. N. Bergmans

**Affiliations:** 1 Department of Dermatology/ Robert H. Lurie Comprehensive Cancer Center, Northwestern University, Chicago, IL USA; 2 Division of Molecular Oncology & Immunology/ Netherlands Cancer Institute, Amsterdam, Netherlands; 3 Department of Surgery, Loyola University Chicago, Maywood, IL USA; 4 National Institute for Health and the Environment (RIVM), Bilthoven, Netherlands; 5 Departments of Dermatology, Microbiology and Immunology, Northwestern University at Chicago, Lurie Comprehensive Cancer Center, Rm 3-121, 303 East Superior Street, Chicago, IL 60611 USA

**Keywords:** Transgenic T cells, CD4, CD8, Placenta, Breastmilk, Risk assessment, Offspring

## Abstract

Tumor immunotherapy using gene-modified T cells has already met with considerable success in the treatment of metastatic melanoma and B cell lymphoma. With improving patient prognoses, new questions arise. In particular, the long-term consequences of treatment among individuals of childbearing age could now be considered. Former patients can carry a cohort of transgenic memory T cells long after treatment has ceased and the effector T cell population has contracted. When patients become parents well after treatment is completed, expectant mothers may still pass transgenic T cells to their unborn children. Consequences should be more measurable if the mother also breastfeeds the baby. Maternal T cells may shape immune responses in the child, can tolerize the child to maternal antigens, and might cause either beneficial or adverse effects in the offspring. The hypothesis put forth is that transgenic T cells transferred from mother to child during and after pregnancy might have consequences that have not been adequately considered to date. Depending on the targeted antigen and the MHC eventually required to present it, such transfer may be beneficial, uneventful or even damaging. Such potential consequences are addressed in this paper. The transgenic T cells might form a pocket of memory T cells in secondary lymphoid organs of the child, expand upon antigen stimulation, and react. However, simple measures might be devised to avoid any reason for concern. These considerations provide ample incentive to probe transgenerational transfer of transgenic T cells.

## Introduction

The hypothesis put forth in this paper is that transgenic T cells adoptively transferred into a woman during therapy may, during and after a future pregnancy, be passed to her unborn child and that these T cells could have consequences for the child. The magnitude of this event depends on the odds that such T cells are transferred and on the opportunities for such T cells to interact with tissue cells in the new host. The consequences may be desirable, or not. This paper is meant to initiate a discussion about the potential transfer of transgenic T cells during pregnancy and by breastfeeding and focuses on its potential implications, and possible interventions.

### Transfer across the placenta

Cells, including T cells, are transferred from mother to child by crossing the placenta during pregnancy [1] as well as by subsequent breastfeeding [2]. Both means of transfer lead to maternal microchimerism. These cells and associated maternal antigens induce Treg development and tolerance to non-inherited maternal antigens (NIMAs) in their new host [1]. This process results in the presence of roughly one in 5000 maternal T cells in an adult [2, 3]. Transplacental migration of maternal T cells was definitively demonstrated using radiolabeled Th1 and Th17 cells in mice [3]. Transmigration increased under inflammatory conditions, suggesting a role in protecting the fetus from harm. This is most dramatically observed in children with SCID, protected in part by maternal T cells [4]. The phenotypes of these transplacental T cells are not a direct reflection of the phenotypes observed in the maternal circulation [5]. As a consequence of NIMA transfer, the child will develop immune tolerance to maternal antigens, including HLA. Interestingly, this should ultimately allow for reduced tissue rejection in children accepting tissue donations from their mother. Favorable outcomes upon tissue donation is just one example of the general impact of maternal T cells on the development and maturation of the child’s immune system.

### Transfer via breastmilk

The transfer of T cells via breastmilk has been demonstrated in several animal studies, and has also been studied in human breastmilk. The colostrum includes a particularly high density of maternal immune cells [2] and from the existing literature, one can extract a fair estimate of 10^7^ T cells per 150 ml of human breastmilk, the majority of which are effector memory T cell subsets [2]. A substantial proportion of T cells are CD4^+^, which can accumulate in lymphoid organs including the Peyer’s patches, spleen, and thymus [6]. An initially large cohort of maternal CD8^+^ memory T cells is also present, and declines following weaning [6]. Transferred T cells educate immune responses in the offspring, such that offspring of immunized mothers develop T cell responses to the immunogen among their own T cells [6]. This evolutionarily refined mechanism is considered important for tolerance induction and for prevention of autoimmune disease in the child. Meanwhile the importance of lactational, immunosuppressive Treg transfer is emphasized by functional immunosuppression independent of MHC (mis)-matching between mother and mouse pups [7]. An example is found in the development of tolerance to NIMAs [2]. To date however, the transfer of natural T cells has not been examined for its ability to mediate cytotoxic, autoimmune side effects. Indeed, if autoimmune disease does develop in the child, a connection to maternal T cells cannot easily be made.

### Relative contribution of placental and lactation transfer

Transgenic T cells can be transferred during pregnancy and by breastfeeding. However, an understanding of the relative contribution of T cells from either source to immune education in the child awaits further studies. A contribution for breastfeeding has been largely overlooked to date, despite this mechanism likely having distinct consequences of its own. Indeed, breastfeeding specifically mediates prolonged immune development and education in the infant mediated through the gut of the newborn and reports suggest that progenitor cells/stem cells in breastmilk can give rise to differentiated cells in the offspring and thereby repair damage, if such exists [2]. This is true particularly because postnatal T cells arrive in a gastro-intestinal environment receptive to immune education.

### Transgenic T cell transfer

The role of maternal T cells in immune education raises concerns regarding the potential transfer of transgenic T cells after cancer immunotherapy. This may also depend on the construct used. T cell receptors (TCRs) react to cognate antigen, but only when presented in the context of matching MHC. There is a 50% chance that the MHC restriction of transferred TCR transgenic T cells will match the child’s tissue. This restriction does not apply to CAR-transgenes, which recognize their target independent of MHC. One might argue that the number of T cells transferred is insufficient to be consequential, yet clinical observations would provide a counterargument. In patients treated with CD19 CAR T cells, the treatment caused B cell aplasia that continued well beyond the presence of detectable CD19 CAR T-cells in the circulation [8]. These data suggest a functional persistence of CD19 CAR T cells at levels below the limits of detection by flow cytometry, while providing incentive for evaluating persistence by q (RT)-PCR instead. Meanwhile, TCR transgenic T cells exhibit great resilience in the host, and a readily measurable amount of transgenic T cells is detected 1 year after myeloablation and adoptive transfer [9]. These cells were readily reactivated in presence of high-dose IL-2. Importantly, transfer of a single central memory T cell is enough to populate the host upon infection-mediated expansion in mice [10]. Thus a similar expansion is likewise possible upon antigen encounter in patient offspring, potentially contributing to prevention of B cell ALL by unintended transfer of one or more transgenic T cells. Yet in cases where transgenic T cells target developmental gene products, some caution may be in order. This can re-ignite the discussion about a need to include a suicide switch such as inducible caspase-9 to allow for a recall in case any adverse events are noted in the offspring. Before such treatment is even considered, one must be aware of the possibility of transgenerational T cell transfer, as it is currently unclear whether these switches can be safely applied during development.

**Fig. 1:**
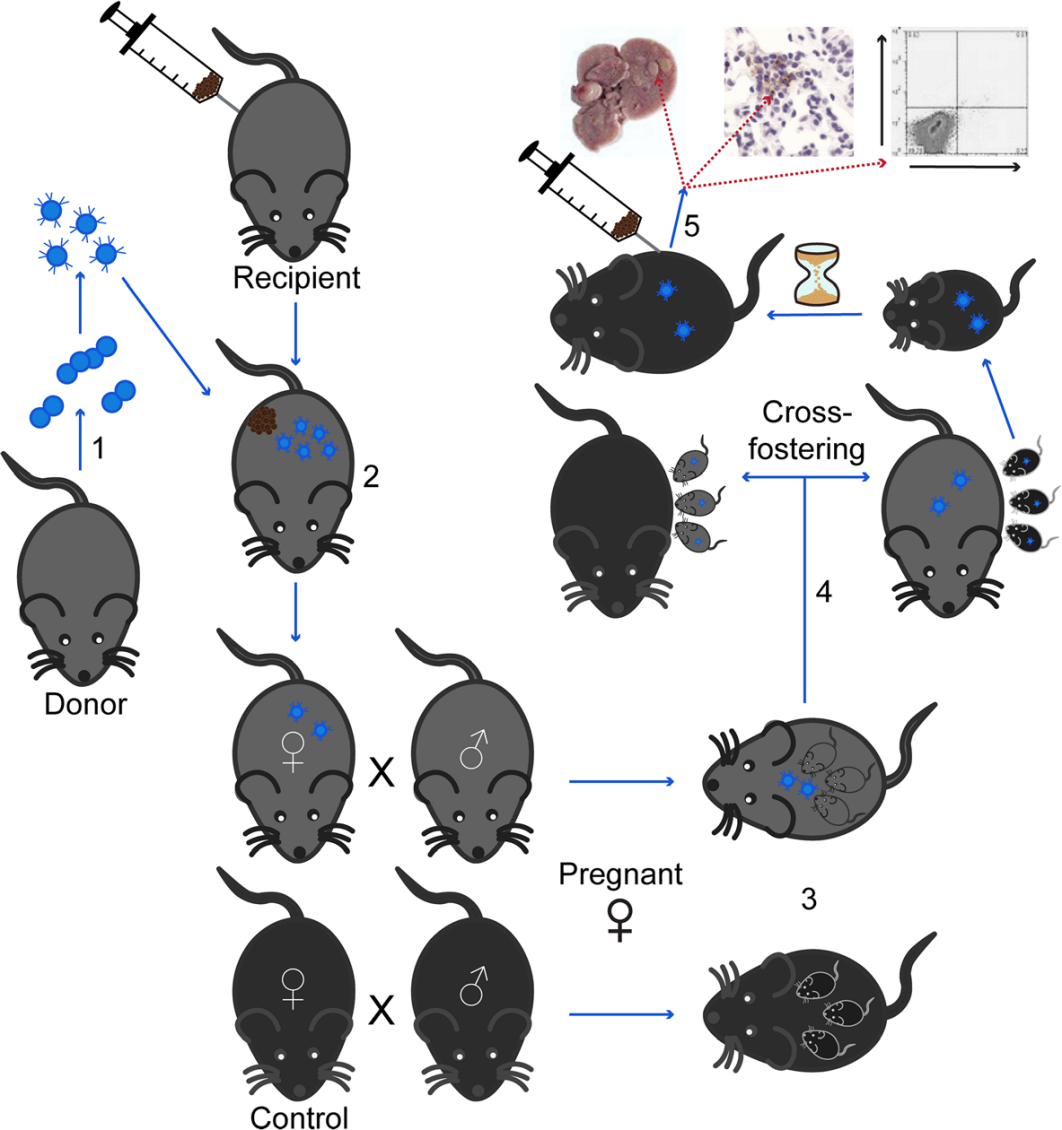
Evaluating transgenerational transfer of adoptively transferred T cells. (1) T cells are isolated from splenocytes and transduced to express a transgenic CAR. (2) Female mice are tumor challenged and treated with CAR T cells, then followed for tumor resolution. (3) After varying intervals, these females or wild type females are bred to have offspring which are (4) then cross fostered. (5) Pups are weaned and challenged with tumor cells or not, then evaluated for tumor growth, autoimmune responses and persistence of transgenic T cells

Transgenerational transfer of therapeutic T cells and its impact on offspring can be studied in mice (Fig. 1). To this end, TCR and/or CAR transgenic T cells would be used to treat healthy or tumor-challenged females, which would then be entered into breeding. The pups would be tested for transgenic T cells upon weaning, in part after being fostered by females who did not receive transgenic T cells, allowing for an understanding of the role of placental vs breastmilk transfer. Depending on the transgene used, studies in the offspring would focus on transgenic T cell prevalence and location, developmental abnormalities, protection from tumor growth and autoimmunity. Such studies would enhance our understanding of the biology of adoptively transferred T cells while ideally laying to rest any concerns about long term consequences of adoptive T cell treatment in female survivors. In a worst case scenario, where risk to newborns were detected, information on how to minimize or ablate these risks through well-informed and researched approaches to breastfeeding could be gleaned.

### Current guidelines

The current product insert for FDA-approved axicabtagene ciloleucel therapy mentions a dearth of knowledge regarding the impact of therapy on pregnancy and lactation, and on future children. This is accompanied by a statement regarding a projected risk for fetal toxicity if cells cross the placenta, based on the mechanism of action of these T cells. It is explicitly stated that no information is available regarding fetal transfer or reproductive or developmental toxicity to the fetus, while the effects on breastmilk production or on a breastfed infant also remain unknown. Similarly, among the manifold of currently active clinical trials, pregnancy or lactation are common exclusion criteria. Importantly, contraception is advised during the period of therapy for approved drugs, and commonly required for participation in clinical trials. The required duration of contraception following treatment greatly varies between trials. Together, these guidelines demonstrate the knowledge gap surrounding the longevity and persistence of CAR-T cells in the patient and concerns for their impact during a future pregnancy. Of course, such concerns could be allayed by incorporating a mechanism to ablate any remaining CAR-T cells, such as a suicide gene, which could be activated should a women choose to conceive after treatment. However, as it is not known how long CAR-T cells must persist in the body to prevent a relapse, their deletion could carry an inherent risk to the woman.

### Ethical and regulatory considerations

A woman undergoing treatment with transgenic T cells should be appropriately counseled about the potential risks to her unborn children, should she wish to conceive at any point following treatment. This warrants a thorough risk assessment in models of disease. Such risk is defined by the chance of an adverse event occurring multiplied by the magnitude of its effect. We can and should appropriately counsel women about future reproductive choices, perhaps discouraging them from breastfeeding their newborn, or in cases where hereditary tumors come into play now or in the future, advising them that children might rake the benefit of maternal T cell therapy if transgenerational therapeutic T cells can be reactivated in the new host. It will be important to test this hypothesis in detail in order to provide patients with a well-informed choice. Aspiring mothers should be counseled based on the results of an informative risk assessment study. At our current level of knowledge, we should be prepared to address the transgenerational consequences of adoptive T cell therapy.

## Data Availability

Not applicable

## Abbreviations

NIMA: Non-inherited maternal antigens
TCR: T cell receptor
